# Preparation of Clindamycin Hydrochloride Loaded De-Esterified Low-Methoxyl Mango Peel Pectin Film Used as a Topical Drug Delivery System

**DOI:** 10.3390/polym12051006

**Published:** 2020-04-27

**Authors:** Tanpong Chaiwarit, Pornchai Rachtanapun, Nutthapong Kantrong, Pensak Jantrawut

**Affiliations:** 1 Department of Pharmaceutical Sciences, Faculty of Pharmacy, Chiang Mai University, Chiang Mai 50200, Thailand; tanpong.c@gmail.com; 2 Division of Packaging Technology, School of Agro-Industry, Faculty of Agro-Industry, Chiang Mai University, Chiang Mai 50100, Thailand; pornchai.r@cmu.ac.th; 3 Cluster of Agro Bio-Circular-Green Industry (Agro BCG), Chiang Mai University, Chiang Mai 50100, Thailand; 4 Department of Restorative Dentistry, Faculty of Dentistry, Khon Kaen University, Khon Kaen 40002, Thailand; 5 Research Group of Chronic Inflammatory Oral Diseases and Systemic Diseases Associated with Oral Health, Faculty of Dentistry, Khon Kaen University, Khon Kaen 40002, Thailand

**Keywords:** pectin, mango, Nam Dokmai, de-esterification, thin film

## Abstract

In this study, we aimed to develop a low-mexthoxyl pectin (LMP) from mango peel pectin through a de-esterification method for use as a film forming agent. The prepared de-esterified pectin (DP) was compared to commercial LMP (cLMP) which possessed a 29% degree of esterification (DE). Mango peel pectin was extracted from ripe Nam Dokmai mango peel using the microwave-assisted extraction method. Pectin derived from the mango peel was classified as a high mexthoxyl pectin (79% DE) with 75% of galacturonic acid (GalA) content. A de-esterification experiment was designed by central composite design to plot the surface response curve. Our prepared DP was classified as LMP (DE 29.40%) with 69% GalA. In addition, the Fourier-transform infrared spectrophotometer (FTIR) spectra of the DP were similar to cLMP and the pectin backbone was not changed by the de-esterification process. Strikingly, the cLMP and DP films showed non-significant differences between their physical properties (*p* > 0.05) with respect to the puncture strength (13.72 N/mm^2^ and 11.13 N/mm^2^ for the cLMP and DP films, respectively), percent elongation (2.75% and 2.52% for the cLMP and DP films, respectively), and Young’s modulus (67.69 N/mm^2^ and 61.79 N/mm^2^ for the cLMP and DP films, respectively). The de-esterified pectin containing clindamycin HCl (DPC) and low-methoxyl pectin containing clindamycin HCl (cLMPC) films demonstrated 93.47% and 98.79% of drug loading content. The mechanical properties of the cLMPC and DPC films were improved possibly due to their crystal structures and a plasticizing effect of clindamycin HCl loaded into the films. The DPC film exhibited a drug release profile similar to that of the cLMPC film. Our anti-bacterial test of the films found that the cLMPC film showed 41.11 and 76.30 mm inhibitory clear zones against *Staphylococcus* *aureus* and *Cutibacterium* *acnes*, respectively. The DPC film showed 40.78 and 74.04 mm clear zones against *S.* *aureus* and *C.* *acnes*, respectively. The antibacterial activities of the cLMPC and DPC films were not significantly different from a commercial clindamycin solution. The results of this study suggest that mango peel pectin can be de-esterified and utilized as an LMP and the de-esterified pectin has the potential for use as a film forming agent, similar to cLMP. In addition, the remarkable use of de-esterified mango peel pectin to prepare films, as shown by our study, holds a great promise as an alternative material for anti-bacterial purposes.

## 1. Introduction

Pectin is a natural polymer obtained from wasted fruit material, such as apples, and bananas, oranges, as well as mangos [1]. In Thailand, there are several mango cultivars, including Nam Dokmai, Mahachanok, Khaio Sawoei, and Okrong Thong. Nam Dokmai mango originated from Thailand and its taste is the most favorable when ripe. It is a major cultivar of commerce in Asia with substantial planting, consumption, and processing [2]. Thus, there is a huge amount of domestic Nam Dokmai peel waste in Thailand. Generally, types of pectin can be categorized by the degree of esterification (DE). Pectins with a DE level over 50% are considered as high-methoxyl pectins (HMP).

**Figure 1:**
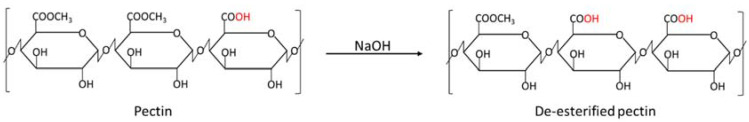
The de-esterification reaction of pectin using NaOH.

HMP gel formation requires acid and dissolved solids, i.e., sugars. However, HMP cannot form gels by interaction with calcium ions due to an insufficient number of carboxylic groups. Pectin with DE levels lower than 50%, categorized as low methoxy pectin (LMP), can form gels by reacting with calcium ions to form egg-box-like structures [3]. LMP can be produced by the de-esterification of HMP using either acidic or basic chemicals. The main disadvantage of acid de-esterification is the slow onset of the reaction. However, this might be accelerated by increasing the temperature, although it may degrade pectin. Alkaline de-esterification exhibits a faster reaction rate than acid de-esterification, but the rate of de-polymerization may be faster than the rate of de-esterification when the temperature is increased [4]. The reaction of the de-esterification process induced by alkaline (NaOH) is shown in Figure 1.

In the field of pharmaceutical sciences, pectin can be used as a film forming agent for dermal and transdermal drug delivery systems, such as thin films. These drug delivery systems exhibit several advantages over other conventional dosage forms in terms of the avoidance of first-pass metabolism, patient compliance, and the ease of applications [5]. The utilization of produced thin films for medical treatments, specifically on human skin, could be diversified, depending on the antibiotics added into the film to target a specific bacterial species.

Clindamycin is an effective antibacterial agent against staphylococci, streptococci, and pneumococci, most anaerobic bacteria, *Chlamydia trachomatis,* and some protozoa. Furthermore, clindamycin exhibits bacteriostatic activity by inhibiting 50s ribosomal activity, thereby inhibiting bacterial protein synthesis. Due to the activity against *Staphylococcus aureus*, streptococci, and anaerobes, clindamycin was found to be effective for the treatment of various skin and soft tissue infections [6]. In addition, clindamycin exerts an effective anti-microbial property against *Cutibacterium acnes,* previously named *Propionibacterium acnes* [7]. Thus, the infusion of clindamycin into anti-acne products has been a laboratory strategy used so far to implement its bactericidal properties.

This study aimed to evaluate the experimental protocol to prepare low-methoxyl pectin, with different methods using basic de-esterification. In addition, the obtained de-esterified pectin (DP) was used as a film-forming agent and the DP films were characterized for their morphological appearance and mechanical properties. Clindamycin HCl-loaded DP films were characterized morphologically and tested for their tensile properties. In addition, the antibacterial activity and clindamycin release profile were investigated.

## 2. Materials and Methods

### 2.1. Materials

Non-amidated low methoxy pectin (LMP; Unipectine OF300C; DE = 29% and DA = 0%) was purchased from Cargill^TM^ (Saint Germain, France). *m*-Hydroxy diphenyl reagent (85% assay) and standard *d*-galacturonic acid monohydrate (≥97.0% assay) were purchased from Sigma-Aldrich (Saint Louis, MO, USA). Clindamycin hydrochloride (HCl) (100% purity) was purchased from Merck (Damstadt, Germany). Sodium hydroxide (NaOH) (concentration 1.000 N) and hydrochloric acid (HCl) (concentration 1.000 N) were purchased from Ajax Finechem (Victoria, Australis). Tris (hydroxymethyl aminomethane) (>99% assay) was purchased from Fisher chemical (Loughborough, UK). Calcium chloride (CaCl_2_) (≥98% assay) was purchased from Merck (Damstadt, Germany). Commercial clindamycin gel (100 mL containing clindamycin HCl equivalent to the clindamycin base at 1%) was purchased from PRC international (Bangkok, Thailand). All other reagents were of analytical grade.

### 2.2. Collection of Mango Peel

Mango (*Mangifera indica* cv. Nam Dokmai) peel wastes were collected from a local market in Chiang Mai, Thailand, during April to May 2019. Mango peel wastes were washed, cut, and then dried in a hot air oven at 50 ± 2 °C. The dried peel was subsequently ground to make a fine powder using a high-speed food processor and filtered by a sieve number 30 (diameter 0.6 mm) before extraction [8].

### 2.3. Extraction of Pectin from Mango Peels by Microwave-Assisted Method

Pectin was then extracted from the dried mango peel powder using a phase-power control microwave extraction (PC-MHG) system [9]. Ripe mango peel powder was mixed with acidified water, adjusted to pH 1.5 by 1 N HCl, using a ratio of 1 g per 15 mL. The mixture was shaken rigorously to obtain a homogenous liquid mixture that was then placed in microwave oven. The pectin in ripe mango peel was extracted by a microwave operating at 2450 MHz with 500 W of power for 20 min. The extract was centrifuged at 4000× *g* for 10 min. Pectin suspended in the supernatant was precipitated by 95% ethanol and then washed by 95% ethanol five times. Finally, the precipitated pectin was dried in a hot air oven at 40 °C. The extraction yield was calculated using Equation (1):

(1) $$\mathrm{Extraction}\;\mathrm{yield}=\frac{W}{Wo}\times 100$$

where: *W* is the weight of the extracted pectin after drying and *Wo* is the weight of the dried ripe mango peel powder.

### 2.4. De-Esterification Using NaOH

The de-esterification method used central composite design (CCD) using Design-Expert^®^ software version 12 (Stat-Ease, Inc., Minneapolis, MN, USA). The variables were the volume of 1 N NaOH (mL) and temperature (°C) in three different levels. Coded experimental levels of two variables for pectin de-esterification re shown in Table 1. The measured outcome was the degree of esterification (%). Data from repeated independent experiments was analyzed and a graph was plotted to obtain the desirable degree of esterification using Design-Expert^®^ software version 12. Each experiment was performed in three independent replications. The de-esterification method was modified from Hunter and Wicker, 2005 [10]. Briefly, one gram of mango peel pectin was dissolved in 100 mL distilled water and the pH was adjusted to 11. Then, 1 N NaOH was added into the pectin solution. The temperature was controlled in a target range of more or less than 2 °C (Table 1). The reaction was performed until the pH of the pectin solution decreased to 11, and then the pH was maintained at 11 for 3 h. The reaction was stopped by adjusting the pH to 5. Then, we added 5 mL of 95% ethanol and boiled for 5 min. Precipitated, de-esterified pectin was collected using a vacuum filter and washed with 50% ethanol at pH 5.2 and 95% ethanol, respectively. De-esterified pectin was dried, as a final step, in the oven at 40 °C.

### 2.5. Degree of Esterification

The degree of esterification (DE) was determined by the titration method with NaOH. The method was modified from Freitas de Oliveira et al. [11]. Briefly, 50 mg pectin powder was dissolved in 20 mL deionized CO_2_-free water. A 0.1% phenolphthalein was used as the reaction indicator. The pectin solution was titrated with 0.1 N NaOH and the end point was recorded when the solution color was immediately changed to pink. This end point was an initial titer. Then, 10 mL of 0.1 N NaOH was added into the pectin solution. The solution was shaken rigorously and kept at room temperature for 2 h. Then, 10 mL of 0.1 N HCL was added into the solution, at which point the pink solution turned colorless. The titration process was repeated once using 0.1% phenolphthalein added into the solution as an indicator. The solution was subsequently titrated with 0.1 N NaOH and the end point was recorded as the final titer. The degree of esterification was calculated using the following Equation (2):

(2) $$\%\;\mathrm{DE}=\frac{final\;titer}{initial\;titer+final\;titer}\times 100$$

### 2.6. Galacturonic Acid Content

The galacturonic acid (GalA) content in pectin powder was evaluated using the *m*-hydroxyldiphenyl method [11]. Pectin powder was diluted to a 100 µg/mL concentration in water. We added 0.125 M sodium tetraborate in concentrated sulfuric acid into a 0.4 mL pectin powder solution on a crushed ice bath, then heated in a 37 °C water bath for 5 min and placed in a crushed ice bath again to cool down. *m*-Hydroxyldiphenyl reagent, prepared by dissolving 0.5 g of *m*-hydroxyldiphenyl in 100 mL of 0.5% NaOH, was added into the pectin solution. At this point, the solution turned pink in 5 min. The GalA in the solution was analyzed using a UV-Vis spectrophotometer (UV-2450, Shimadzu, Kyoto, Japan) at 537 nm. A standard curve was prepared with the galacturonic acid standard solution at 50–200 µg/mL. The calculated correlation coefficient (*r*^2^) of linear regression was 0.9968.

### 2.7. Fourier-Transform Infrared Spectrophotometry

LMP and de-esterified pectin were identified and evaluated for their functional groups by Fourier-transform infrared spectrophotometer (FTIR) (alpha FT-IR, Bruker, Bermen, Germany). Pectin powder (10 mg) was homogenously mixed with 100 mg of dry KBr powder. Then, the mixture was placed on an FTIR plate and inserted into the instrument for reading. The spectra were recorded at 3000 to 600 cm^−1^ transmittance mode at 4 cm^−1^ resolution. The blank control used for the assay reading was a dry KBr power without pectin. The data were integrated with OMNIC software (Bruker, Bermen, Germany) for an analysis.

### 2.8. Film Preparation

Modified ionotropic gelation with a solution-casting method [12] was selected to prepare the pectin film. Briefly, a pectin solution was prepared by dissolving 3 g of pectin powder in 100 mL deionized water and the crosslinking solution was CaCl_2_ 3% *w*/*v*. Pectin solution (3 mL) was evenly dropped on the dialysis membrane (Cellu-Sep T3/Nominal MWCO: 12,000–14,000 Da, Membrane Filtration Product, Inc., Texas, USA), fixed with a 6 × 6 cm plastic case. Then, the membrane was placed on the CaCl_2_ solution and allowed to stand for 10 min. The cast film was transferred from the dialysis membrane to a plastic box and dried in the oven at 40 °C for 24 h. Finally, the dried films were wrapped in silicone paper and aluminum foil and kept at room temperature until further analysis.

### 2.9. Analysis of Morphological Characteristics

The morphology of the thin film was evaluated for its thickness and surface texture by utilizing a scanning electron microscope (JSM-5410LV, JEOL USA, Inc., Peabody, MA, USA). The experiment was conducted under a low vacuum mode at 10 kV. The films were inspected under SEM without any coating. The film surface and thickness were observed at 500 and 340 magnification levels, respectively.

### 2.10. Test of Mechanical Properties

**Figure 2:**
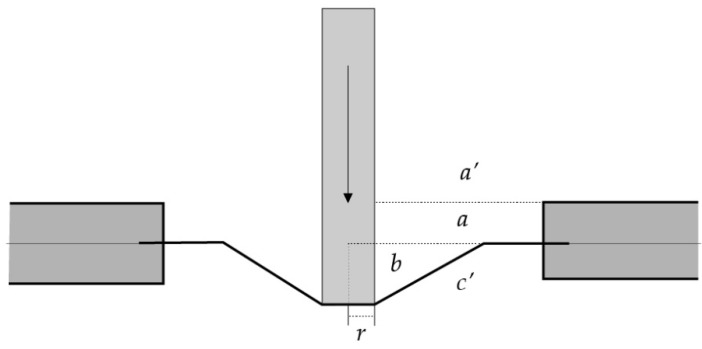
Determination of elongation using a plane flat-faced surface probe, where *a* is the radius of a film in the sample opening holder, *b* is the distance of the probe displacement, *r* is the probe radius and *a’* is the difference between the radius of the film and the probe radius (a–r) [14].

The thin films were cut into square shapes (2 × 2 cm). The film thickness was measured using a thickness gauge (GT-313-A, Gotech testing machines Inc., Taiwan). The tensile properties were evaluated with a texture analyzer TX.TA plus (Stable Micro Systems, Surrey, UK) with 5 kg load cell (0.001 N of sensitivity) used in combination with a compression probe—a 2 mm diameter plane flat-faced surface probe. In short, the film samples were fixed on a stand to achieve a 20 mm distance between the probe head and the samples (Figure 2). The test was operated using a probe speed of 2 mm/sec [13]. The data, i.e., the time (s), force (N), and distances (mm), were recorded when the probe was in contact with the film surface. Similar independent experiments were repeated five times. The characterization of the mechanical properties included the puncture strength, percent elongation at the break, and Young’s modulus which were calculated by Equations (3)–(5), respectively [14,15].

(3) $$\mathrm{Puncture}\;\mathrm{strength}=\frac{Fmax}{A}$$

where *Fmax* is the force at the film break point (N) and *A* is the film surface area in contact with the probe surface (mm^2^).

(4) $$\mathrm{Percent}\;\mathrm{elongation}\;\mathrm{at}\;\mathrm{break}=\left(\frac{\sqrt{{a^{\prime }}^{2}+b^{2}}+r}{a}-1\right)\times 100$$

(5) $$\mathrm{Young}’s\;\mathrm{modulus}=\frac{slope}{Film\;thickness\times probe\;speed}$$

where the slope is obtained from the plotted graph between force (N) and time (s).

### 2.11. Preparation of Film Containing Clindamycin HCl

Solutions (3% *w*/*v*) of either cLMP or DP were prepared by dissolving pectin powder in de-ionized water. The clindamycin HCl equivalent to clindamycin base 1 g was added into 100 mL of pectin solution to obtain 1% *w*/*v* clindamycin in a film forming solution. Films containing clindamycin HCl were prepared by modified ionotropic gelation with the solution-casting method [12]. The prepared solution was dropped on a dialysis membrane (Cellu-Sep T3/Nominal MWCO: 12,000–14,000 Da, Membrane Filtration Product, Inc., Texas, USA), fixed with a 6 × 6 cm plastic case, and cast on 3% *w*/*v* CaCl_2_ for 10 min. The cast film was removed from the dialysis membrane and dried in an oven at 40 °C for 24 h.

### 2.12. Clindamycin Content

Film portions (2 × 2 cm) were cut from the dried film containing clindamycin HCl (6 × 6 cm). The thickness and weight of each portion were measured. The film (2 × 2 cm) was completely dissolved in 5 mL of 0.1 M Tris (hydroxymethyl aminomethane) (tris) buffer solution pH 7.4. The solution was analyzed using a UV-spectrophotometer (UV2450, Shimadzu, Kyoto, Japan) at 211 nm. The clindamycin HCl content was calculated from the referenced standard curve prepared with a clindamycin HCl solution in a concentration range of 0.05–0.4 mg/mL. In each experiment, we obtained a standard curve with a high linear regression (data not shown). Each film sample was tested in triplicate. The clindamycin HCl content of the tested films was calculated with the following Equation (6)

(6) $$\mathrm{Clindamycin}\;\mathrm{HCl}\;\mathrm{content}\;(\%)=\frac{Amount\;of\;entrapped\;drug}{Theorectical\;drug\;content}$$

### 2.13. In Vitro Drug Release Profile

The analysis of the in vitro drug release profile was performed as previously described [13]. Films containing clindamycin HCl in a square shape (2 × 2 cm^2^) were immersed in 15 mL Tris buffer pH 7.4 at 32 ± 0.5 °C in semi-static conditions. The dissolution media (3 mL) was taken at predetermined times (1, 3, 5, 15, 30, and 60 min) and the Tris buffer (3 mL) was replaced. The samples were analyzed for clindamycin HCl release using a UV-spectrophotometer (UV2450, Shimadzu, Kyoto, Japan) at 211-nm wavelength. All dissolution experiments were performed in triplicate.

### 2.14. Antibacterial Activity Test

The antibacterial activity test was performed, similar to a recent study [16]. Stock cultures of *Staphylococcus aureus* (ATCC25923) and *Cutibacterium acnes* (ATCC6919), previously named *Propionibacterium acnes*, were growth in tryptic soy agar (HiMedia, Mumbai, India) at 37 °C for 24 h., and subsequently inoculated into tryptic soy broth (Sigma-Aldrich, St. Louis, MO, USA) and grown at 37 °C under an aerobic atmosphere for 12 h. Overnight cultures of *S. aureus* and *C. acnes* were determined for the optical density at 600 nm wavelength (OD600) using a spectrophotometer (Beckman Coulter, Fullerton, CA, USA) right before a fresh preparation of bacterial stocks.

All tested film samples were sterilized by ethylene oxide prior to an antibacterial test. The agar diffusion method was used to assess the films’ ability to kill two tested skin pathogens by their clindamycin content. Briefly, 100 µL bacterial stock (OD600 = 0.1) was plated on dried tryptic soy agar plate and let dried. The tested film disks (0.4 mm diameter) were placed on the prepared agar plate and left in a 37 °C aerobic incubator for 16–18 h. A zone of inhibited bacterial growth, seen as a clear zone, was measured using a Mitutoyo^®^ Digimatic caliper (Mitutoyo Corporation, Kanagawa, Japan). The bacterial growth under the film disks was also investigated. Films without clindamycin HCl were used as a blank control. Furthermore, a Whatman^®^ antibiotic assay disc (GE Healthcare, Pittsburgh, PA, USA) loaded with 10 µL clindamycin solution (Clinda-M, RPC International Co., Ltd., Bangkok, Thailand) equivalent to 1% clindamycin base served as a positive control.

### 2.15. Statistical Analysis

The represented data were expressed as the mean ± standard deviation (S.D.) The significance of the results was analyzed by SPSS software (version 17; IBM Corporation, Armonk, NY, USA). A significant level (*p*-value) of less than 0.05 was considered statistically different. The software used to evaluate the experimental design was the Design-Expert program (version 12; State-Ease Inc., Minneapolis, MN, USA).

## 3. Results and Discussion

### 3.1. Mango Peel Pectin Extraction and Characterization

The extraction yield and chemical characteristics of the mango peel pectin are shown in Table 2. We found from our extraction method that the % yield of the mango peel pectin obtained from microwave-assisted extraction was 12.46% ± 0.52% *w*/*w*. Our extraction protocol resulted in a pectin yield similar to a previous study which found that extraction of *Mangifera indica* cv. Nam Dokmai gave a pectin yield roughly at 12% *w*/*w* [17]. Furthermore, the yield from the microwave-assisted extraction was higher than the conventional solvent extraction method using an acidified water pH 1.5. Sommano et al. [18] investigated the pectin content in several mango cultivars, including Nam Dokmai mango, and determined that the yield of pectin extracted from Nam Dokmai mango peels using conventional extraction was approximately 0.9% *w*/*w* [18].

Conventional extraction resulted in a low yield as pectin might be degraded under a long exposure to high temperatures. In addition, microwave heating was better than conventional heating, as microwave energy generates heat inside the material instantly and rapidly from water molecule re-orientation during the heating process, thereby resulting in a heat and mass gradient transferring from the material to the solvent [19,20]. The GalA content of the obtained mango peel pectin was 75.14% ± 5.09%. This content is an important characteristic of pectin as GalA is indicative of the pectin content and the purity of the pectin molecule. A higher GalA content attributes to a higher pectin content in extracted pectin powder [21].

Although this study did not focus on an optimal condition and effect of extraction factor, Pandit et al. [21], whose study was to evaluate the influence of microwave power and extraction on extracted mango (*Mangifera indica* cv. Totapuri) peel pectin, found that microwave power enhanced the penetration of the solvent into the cellular matrix. However, high power, i.e., at 1000 W, decreased the extraction yield, possibly due to the degradation of pectin at high temperatures. The quality of the extracted mango peel pectin was also regulated by the extraction time, as the extraction yield and GalA content were increased when longer extraction times (20 min) were employed. However, the GalA content was decreased after 25 min of extraction time, possibly due to pectin degradation. In addition, the pectin content in mango peel was found to depend on the fruit ripening [22]. The degree of esterification (DE) of the obtained mango peel pectin in this study was about 79%, and thus classified as a high methoxyl pectin (HMP). In another study, the extracted pectin from ripe mango peels without a de-esterification process was also classified as HMP with a DE of 62–86% [17]. Therefore, the variation of DE between extraction batches depends on the difference between the degree of mango ripeness as well as the extraction methods.

### 3.2. Experimental Design

The degree of esterification under a desired variable, an actual and predicted value of DE are shown in Table 3. In this experiment, quadratic analysis was a suitable model for the study. The predicted R^2^ of 0.9026 was in reasonable agreement with the adjusted R^2^ of 0.9615 (the difference was less than 0.2). The result obtained from analysis of variance (ANOVA) showed a statistically significant difference of coefficients of temperature and volume of NaOH between groups (*p* < 0.0001), as shown in Table 4. The results showed that both high temperatures and increasing the volume of 1 N NaOH, within the range of study, led to a decrease of DE. The effect of temperature × volume of NaOH was not significant.

**Figure 3:**
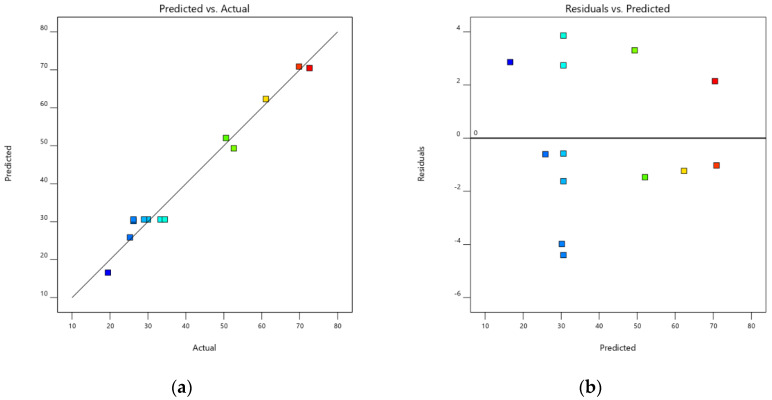
Graph plotted between the predicted and actual degree of esterification (DE) (**a**) and plot of the residuals versus the predicted DE of de-esterified pectin sample (**b**).

However, the experiment performed at 25 °C was easier to control than that performed at lower temperature and the added volume of NaOH at 25 °C was shown to obtain a desirable DE. Even though the model was significantly different, the lack of fit was not significant. An adequate precision of more than 4 was desirable and indicates that the model can be used to navigate design space [23], thus our adequate precision of 22.3518 suggests that our experiment yielded a high precision. This might result in a low level of pure error as shown in Table 4. The graph plotted between the actual and predicted DE showing a high correlation coefficient (R^2^ = 0.9775) indicates that this model has a good fit Figure 3.

**Figure 4:**
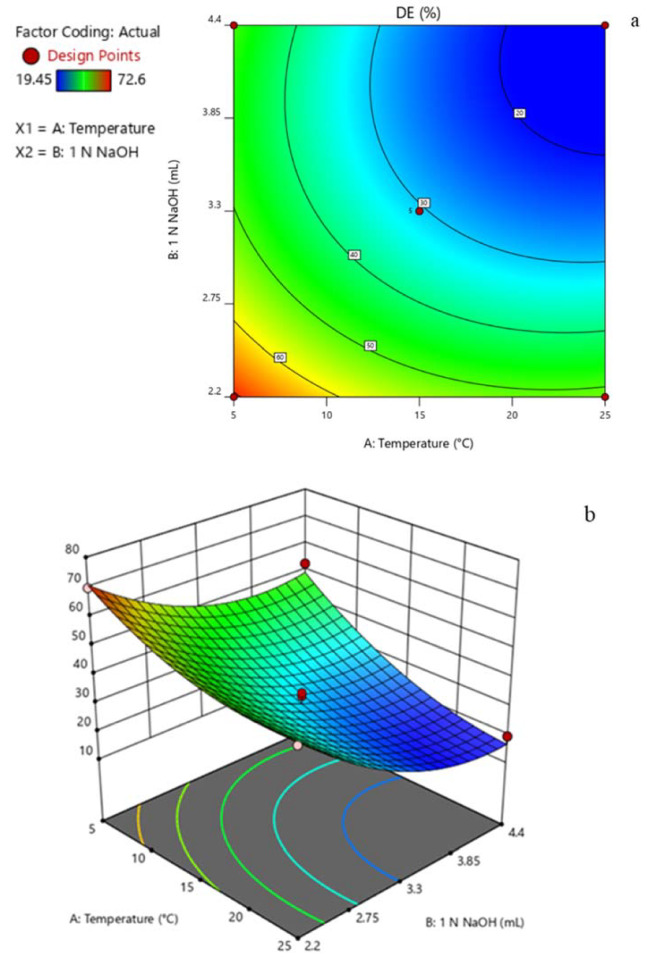
Response surface showing the effect of the temperature and volume of 1 N NaOH in a level of experiment on DE of de-esterified pectin. (**a**) contour response; (**b**) 3D response.

The residuals plot between the residuals and the predicted DE value did not appear to show an association Figure 3. This indicates that the normality, independence, and randomness of the residuals were satisfactory [24]. From the model fitting, the DE value can be predicted from Equation (7) as well as the response surface obtained from quadratic regression model Figure 4. The coefficients of Equation (7) and the response surface were obtained from investigating the coded level (Experiment order 1–13) in Table 3. The different colors in Figure 3 and Figure 4 indicated a range of group of response (% DE) as shown in Table 3. Red and blue indicated high and low DE, respectively. Briefly, red color indicated DE around 70%, yellow color was around 60% DE, green color was around 50% DE, and blue color was around 20% DE. The response surface showed that an increase of temperature and/or volume of NaOH reduced the DE value. To obtain a DE of 29%, equivalent to that of a commercial LMP, the reaction should be operated at 25 °C and a volume of 3.05 mL of 1 N NaOH should thus be added.

The comparison between the actual and predicted DE obtained from the tested conditions is shown in Table 3. A statistical *t*-test investigating 13 experiments at a significance level of 0.05 showed a negligible difference between the actual and predicted values. A standard deviation of the predicted DE, obtained from the model, was 3.57%. In addition, the experiment performed under 25°C using 3.05 mL of NaOH yielded 29.40% ± 3.58% DE, which was not statistically significant when compared to the predicted DE (*p* < 0.05). This indicates that this model can be used to predict the DE value from the de-esterification process. De-esterified pectin (Exp. order “DP” in Table 3), obtained from this condition, was then characterized and prepared for thin films to compare with the commercial LMP.

DE = 180.91239 − 2026846T − 61.98791V − 0.316591TV + 0.067487T^2^ + 8.14979V^2^. (7)

where T is the temperature (°C) and V is the volume of 1 N NaOH (mL).

### 3.3. De-Esterification of Pectin

De-esterified pectin (DP), obtained under 25 °C with the addition of 3.05 mL 1 N NaOH (Table 3), was classified as LMP with 29.40% DE. This pectin contained galacturonic acid 69.15% ± 1.19% *w*/*w*. The % yield of DP was 76.72% ± 4.22% *w*/*w*, which indicated a slight pectin loss under this de-esterification condition. From the surface response curve Figure 4, both the volume of 1 N NaOH and the temperature had an effect on the degree of esterification (DE). The DE was decreased when the volume of 1 N NaOH and/or temperature was increased. In 2005, Hunter and Wicker investigated the effect of NaOH volume at room temperature, regardless of the reaction time and found that a higher NaOH volume at room temperature could produce lower DE pectin [10]. Furthermore, high temperatures could reduce the DE as, under higher temperatures, the operation can accelerate the reaction rate [25].

### 3.4. Fourier-Transform Infrared Spectrophotometry (FTIR)

**Figure 5:**
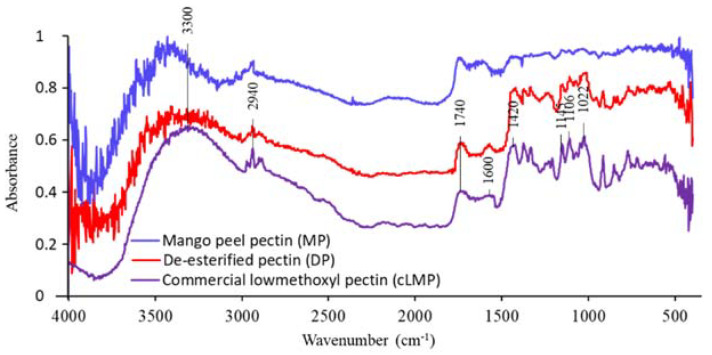
Fourier-transform infrared spectrophotometry (FTIR) spectra of mango peel pectin (MP), de-esterified pectin (DP), and commercial low methoxyl pectin (cLMP).

The FTIR spectra of MP, cLMP and DP are shown in Figure 5. All pectin samples showed similar spectrum patterns. In the fingerprint region around 1200–400 cm^−1^, MP exhibited a similar pattern compared to cLMP but showed a weaker response. When MP was de-esterified to make DP, the fingerprint was clearer and evidently similar to that of cLMP. A board area at approximately 3200 cm^−1^ was caused by the stretching of hydroxy groups (–OH) and a peak at 2940 cm^−1^ occurred from the C–H starching of CH_2_ groups. There were some peaks relating to DE. A peak around 1740 cm^−1^ corresponded to the C=O stretching of methyl ester groups (–COCH_3_), whereas two peaks at around 1600 and 1420 cm^−1^ might be associated with asymmetrical and symmetric stretching of the C=O of carboxylic groups (–COOH). The peaks between 1100 and 1000 cm^−1^ were due to glycosidic linkages between sugar units. [26,27]. In cLMP, a peak of the methyl ester groups (1740 cm^−1^) was weaker than the carboxylic groups (1420 cm^−1^). A similar result was found in the spectrum of DP, in which the ester and carboxylic groups were specified at 1740 and 1420 cm^−1^, respectively. After the de-esterification process, the peak of carboxylic groups (–COOH) still appeared because the reaction did not eradicate all carboxylic groups. In this study, we used a commercial low methoxyl pectin (cLMP; DE = 29%) as our control standard. So, this functional group was not completely removed from DP by de-esterification and we tried to achieve DE as close as 29% for an experimental comparison. Therefore, the DP exhibited same spectra as low methoxyl pectin (cLMP; DE = 29%) and that the DP in FTIR spectra is DP sample in Table 3 which had 29.40% of actual DE value. This result indicated that the de-esterification process did not change the pectin’s backbone structure of the obtained DP comprised of new functional groups.

### 3.5. Morphological Analysis of De-Esterified Pectin Film

**Figure 6:**
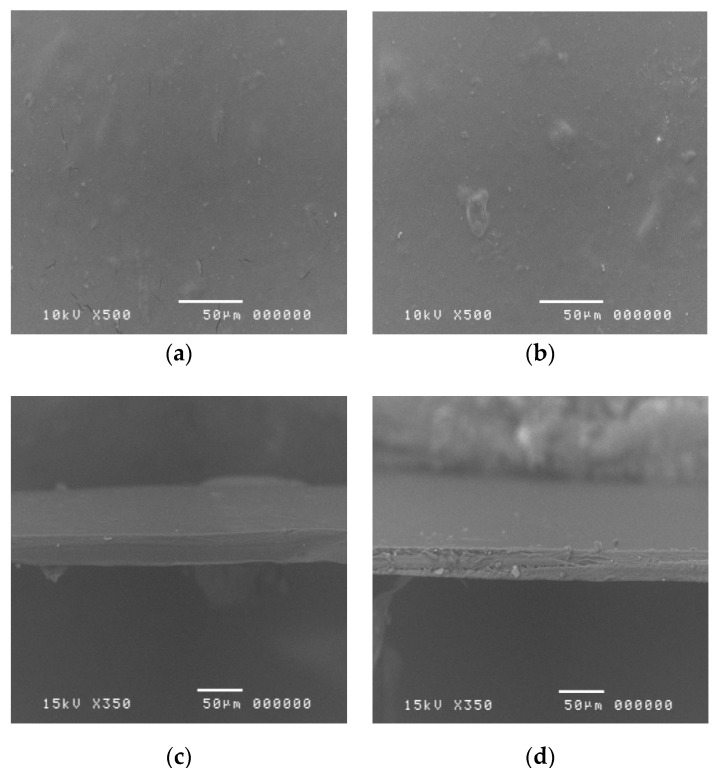
Scanning electron microscopy micrographs of the surface and crossection of cLMP and DP films at 500 and 340 magnification. (**a**) surface of a cLMP film; (**b**) surface of a DP film; (**c**) cross-section image of a cLMP film; (**d**) cross-sectional image of a DP film.

Scanning electron microscopy (SEM) of cLMP and de-esterified pectin are shown in Figure 6. The cLMP and DP films demonstrated a smooth film surface. The thicknesses of the cLMP and DP films measured with a thickness gauge were 0.048 ± 0.0011 and 0.049 ± 0.010 mm, respectively. Significant differences were not observed (*p* > 0.05). In addition, no small white particulate defects, which indicate a non-homogenized polymer matrix, were observed. This result indicates that the cLMP and DP films showed uniformity of the polymer matrix [28].

### 3.6. Mechanical Properties of Prepared Pectin Films

Table 5 showed the mechanical properties of the cLMP and DP films. The mechanical properties of the films were evaluated for several parameters, including the puncture strength, elongation, and Young’s modulus. The puncture strength, a surrogate measurement of film hardness, was a maximum force applied on the film surface at the break point. However, some studies have reported the puncture strength as a measurement of film flexibility [13,14,29,30]. In this study, we used the percent elongation at the break to reflect the flexibility of the films. This defines the ability of a film to deform before the breaking point [31]. The Young’s modulus or elastic modulus refers to the rigidity of the tested materials. Thus, the higher the Young’s modulus value, the more rigid the material is. This is represented as the ratio of stress applied over the strain in the region of elastic deformation [30,32].

In this study, the puncture strengths of the cLMP and DP films were 13.72 ± 3.19 and 11.13 ± 1.92 N/mm^2^, respectively. There was no significant difference between the puncture strengths of the cLMP and DP films (*p* > 0.05). The elongation of the cLMP film (2.75% ± 0.68%) was not significantly higher than that of the DP film (2.52% ± 0.85%) (*p* > 0.05). Similarly, the Young’s modulus of cLMP (67.69 ± 12.26 N/mm^2^) was not significantly higher than that of the DP films (61.79 ± 8.32 N/mm^2^). Thus, we summarize that the mechanical properties of the cLMP and DP films were statistically identical, possibly due to similar DE values. In general, pectin films prepared by the inotropic gelation method with low DE pectin are usually more rigid than films made of higher DE pectin. Higher tensile strengths were observed in lower DE pectin films. However, the elongation was increased with increasing DE, and lower DE pectin films usually exhibited a higher Young’s modulus than higher DE pectin films [33].

### 3.7. Characteristics of Clindamycin HCl-Loaded Pectin Films

The thickness and mechanical properties of cLMP and DP films are shown in Table 5. The thicknesses of cLMP containing clindamycin HCl (cLMPC) and DP loaded with clindamycin HCl (DPC) films were 0.051 ± 0.007 mm and 0.046 ± 0.006 mm, respectively, slightly thinner than their non-loaded corresponding films. The theoretical drug content was calculated directly from the amount of clindamycin HCl added into the pectin solution as the drug completely dissolved in pectin solution. Clindamycin HCl is a water soluble drug and the volume of water in aqueous pectin solution is sufficient for dissolving clindamycin. During the casting process of the film, clindamycin-containing pectin solution demonstrated a clear gel film similar to the blank film (cLMP), suggesting a complete dissolution of clindamycin HCl in the prepared solution.

The clindamycin HCl contents in cLMPC and DPC films were 93.47% ± 4.71% and 98.79% ± 2.69%, respectively. Thus, both cLMPC and DPC films contained a high clindamycin HCl loading content with no significant difference (*p* > 0.05). Clindamycin HCl is water-soluble drug which can dissolve in hydrophilic polymers, like pectin, in aqueous solution, thus explaining the negligible significance of the film thickness compared between the films containing clindamycin HCl and films without clindamycin HCl (*p* > 0.05).

**Figure 7:**
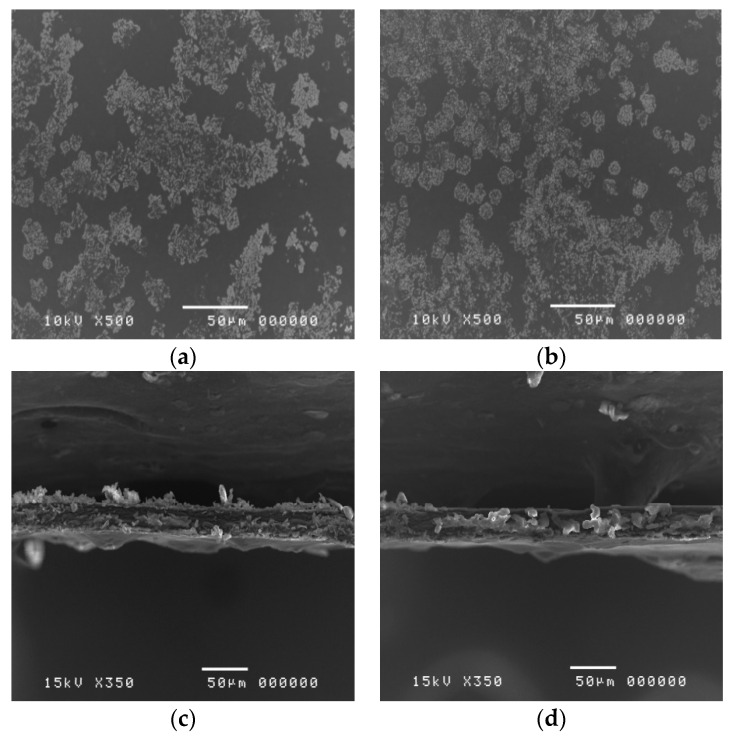
SEM micrographs of the surface and cross section of cLMPC and DPC films at 500 and 340 magnification. (**a**) surface of a cLMPC film; (**b**) surface of a DPC film; (**c**) cross-sectional image of a cLMP film; (**d**) cross-section image of a DPC film.

The SEM images of the cLMPC and DPC films are shown in Figure 7. After the preparation procedure, cLMPC and DPC demonstrated clear films with smooth surfaces. However, when the clindamycin HCl-loaded cLMPC and DPC films were observed under SEM, the crystal structures of clindamycin HCl were visible, despite a complete dissolution of the clindamycin HCl in pectin solution during the preparation. The crystal structure of clindamycin HCl was observed on the surface of both cLMPC and DPC films Figure 7a,b. The cross-sectional images showed a rough texture Figure 7c,d, while the SEM images of cLMP and DPC films (without clindamycin) showed smoother textures Figure 6c,d. These SEM images might indicate that the clindamycin HCl dissolved in the polymer matrix was partially in the form of a crystal structure.

The puncture strengths of the cLPMC and DPC films were significantly decreased (*p* < 0.05) from 13.72 ± 3.19 to 6.78 ± 1.85 N/mm^2^ and 11.13 ± 1.92 to 4.66 ± 0.64 N/mm^2^, respectively when they were compared with the same pectin films without clindamycin HCl as shown in Table 5. When clindamycin HCl was added into the pectin films, the elongations of the cLMPC and DPC films were not significantly changed (*p* > 0.05) (from 2.75% ± 0.68% to 2.46% ± 0.29% and 2.52% ± 0.85% to 2.77% ± 0.44%, respectively), but the Young’s moduli of cLMPC and DPC films tended to decrease significantly (*p* < 0.05) as demonstrated in Table 5. Taken together, the mechanical properties of the cLMP and DP films were likely improved when clindamycin HCl was added.

These findings might be explained by composition of the crystal structure and the plasticizing effect of clindamycin HCl. The incorporation of drugs or other excipients with crystalline or amorphous structures change the morphological state and have an impact on the mechanical properties [15]. Another possible reason to explain our findings was the plasticizing effect of incorporated drugs. Clindamycin HCl has hydroxy (–OH) and amide (–CONH) groups; therefore, it is possible to form hydrophilic interactions, i.e., hydrogen bonds with the hydroxy (–OH), methyl ester (–COOCH_3_), and carboxylic (–COOH) groups of pectin. These interactions disrupt the polymer–polymer interactions, and lead to rearrangement of the polymer chains. A previous study found that ibuprofen exhibited a plasticizing effect and improved the mechanical properties of Eudragit RS^®^ films. The carboxylic (–COOH) group of ibuprofen potentially interacts with the ammonium and ester groups of Eudragit RS^®^. Ibuprofen disrupts an interaction between the polymer chains through hydrogen bonding and this interference results in a plasticizing effect [34].

### 3.8. Drug Release Profile

**Figure 8:**
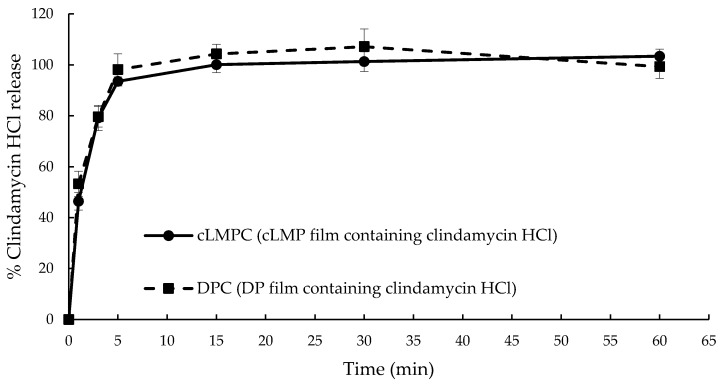
The dissolution profile of pectin films containing clindamycin HCl in tris buffer solution, pH 7.4, at time intervals ranging from 0 to 60 min.

Experiments to determine the in vitro clindamycin HCl release were performed in tris buffer (0.1 M) at 35 ± 0.5 °C. The dissolution profile of the pectin films containing clindamycin HCl is shown in Figure 8. Both the cLMPC film and DPC films exhibited a similar release profile consisting of two step of drug release. The first step might involve the release of clindamycin HCl found on the film surface as seen in the surface morphology. Clindamycin HCl is soluble in water thus it was released immediately when the films contacted water and swelled. From this result, both the cLMPC and DPC films released 93.52% ± 1.83% and 98.12% ± 6.21% of clindamycin HCl in 5 min, respectively, without a detected significant difference (*p* > 0.05). A plateau pattern was observed in the second step. After 5 min, the cLMPC film and DPC films released clindamycin HCl constantly to a 100% level and lasted for the entire test time frame (60 min at least). A significant difference was not observed for all time intervals, possibly because both cLMP and DP exhibited similar DE values. Swelling, dissolution, and degradation of calcium-crosslinking pectin films depended on the DE value [33]. Therefore, it was possible that the cLMP and DP films, with equal DE values, showed non-significantly different percentages of drug release and release profiles.

### 3.9. Activity against Microbial Growth of Clindamycin HCl-Containing Pectin Films

A disc diffusion assay was performed to evaluate the anti-bacterial activity of pectin films containing clindamycin HCl against *S. aureus* and *C. acnes* growth. The positive control was a clindamycin solution (commercial product) containing clindamycin HCl (equivalent to 1% clindamycin base) and pectin films without clindamycin HCl were used as a negative control. The blank film exhibited an inhibitory clear zone due a partial anti-bacterial property of pectin. Pectin showed antibacterial activity against some Gram-positive and Gram-negative bacteria, including *S. aureus* and *C. acne.* In addition, pectin possessed anti-adhesive properties against *S. aureus* and *C. acne* with a MIC of 0.01 mg/mL [35].

The commercially available clindamycin solution, cLMPC and DPC films resulted in a wider inhibitory clear zone against *S. aureus* than *C. acnes* as the clindamycin MIC_50_ value against *C. acnes* is lower than that which kills *S. aureus* (0.047 µg/mL and 0.25 µg/mL, respectively) [36,37]. When the zones of inhibition against *S. aureus* and *C. acnes* growth of cLMPC (41.11 ± 0.33 and 76.30 ± 0.98 mm, respectively) and DPC films (40.78 ± 0.37 and 74.04 ± 1.12 mm, respectively) were compared with blank film (7.18 ± 0.00 and 7.63 ± 0.00 mm, respectively), the cLMPC and DPC films showed a statistically significant wider inhibitory clear zone against the growth of both *S. aureus* and *C. acne* (*p* < 0.05). The antibacterial activity of cLMPC and DPC films against *S. aureus* and *C. acne* were not significantly different (*p* > 0.05) as shown in Table 6.

Clindamycin solution conferred an insignificantly higher anti-bacterial activity (42.02 ± 0.52 and 77.18 ± 1.50 mm for *S. aureus* and *C. acnes*, respectively) compared with the cLMPC and DPC films (*p* > 0.05). This result indicates that the DP extracted from Nam Dokmai Mango peel might be used as a film forming agent for topical antibacterial applications, similar to a commercial low-methoxyl pectin. Mango peel pectin could thus be a promising natural source as an alternative to the clindamycin topical formulation commonly used for the treatment of skin infections.

## 4. Conclusions

In this study, a DP film was de-esterified from mango peel pectin, extracted using the microwave-assisted method. Subsequently, the DP was used to form a thin film. By means of an inotropic gelation with the solution casting method we compared the mechanical properties to cLMP films. A de-esterification process was designed by CCD to plot the surface response curve. The quadratic model was used for analysis showing a high precision correlation coefficient (R^2^ = 0.9775) and no trend in the residue-predicted DE plot. This study investigated two effects, which were the added volume of 1 N NaOH and the operating temperature.

Our results showed that the DE was decreased when a higher volume of 1 N NaOH and/or temperature were employed in the preparation. DP, obtained from 3.05 mL of 1 N NaOH at 25 °C, exhibited 29.40% DE. DP exhibited an FTIR spectra with pectin functional groups and a fingerprint region pattern similar to cLMP. The puncture strength, elongation, and Young’s modulus values of cLMP were not significantly different from the DP film. When clindamycin HCl was incorporated into pectin films, the cLMPC and DPC films exhibited a substantial amount of drug loading content with no significant difference. In term of the mechanical properties, cLMPC and DPC had better puncture strengths and Young’s moduli than the films without clindamycin HCl, possibly due to a crystal structure and the plasticizing effect of clindamycin HCl. In the in vitro clindamycin release study, both cLMPC and DPC exhibited the same pattern of dissolution profile and 100% release of clindamycin HCl in 15 min due to the water soluble properties of clindamycin HCl and the presence of amorphous crystal structures of some part of the incorporated clindamycin HCl. The cLMPC and DPC films conferred an antibacterial activity against *S. aureus* and *C. acnes* to a level comparable to a commercially available clindamycin solution. This study indicated that the DP from Nam Dokmai mango peel held a great potential to be a film forming agent when compared to cLMP. Moreover, it could be used as an alternative treatment agent to replace clindamycin gels. The results from this study can be useful for the further development of films fabricated from natural resources. However, the mucoadhesive properties, skin penetration test, in vivo study, etc., must be carefully examined to ensure efficacy for clinical applications, in relation to the commercially available products.

## Figures and Tables

**Table 1:** Coded experimental level of two variables employed for pectin de-esterification method.

Variables	Level				
	−α	−1	0	+1	+α
1 N NaOH (mL)	1.74	2.20	3.30	4.40	4.86
Temperature (°C)	0.86	5.00	15.00	25.00	29.14

**Table 2:** The chemical characteristics of pectin.

Pectin	Chemical Characteristics		
	Yield (% *w*/*w*)	Galacturonic Acid (% *w*/*w*)	Degree of Esterification (%)
Commercial low methoxyl pectin (cLMP)	-	84.00 ± 3.11	29
Mango peel pectin (MP)	12.46 ± 0.52	75.14 ± 2.09	79.02 ± 2.84
De-esterified pectin (DP)	76.72 ± 4.64	69.15 ± 1.19	29.40 ± 3.58

**Table 3:** The variable levels and responses of degree of esterification-based volume of NaOH and temperature.

Exp. Order	Variables		Response	
			DE (%)	
	1 N NaOH (mL)	Experimental Temperature (°C)	Actual Value	Predicted Value
1	2.20	5.0	69.82 ± 2.55	70.85
2	2.20	25.0	50.57 ± 0.99	52.04
3	4.40	5.0	52.63 ± 1.02	49.33
4	4.40	25.0	19.45 ± 4.81	16.59
5	3.30	1.0	61.08 ± 7.64	62.31
6	3.30	29.0	25.26 ± 3.18	25.86
7	1.74	15.0	72.60 ± 2.73	70.46
8	4.86	15.0	22.73 ± 3.94	30.17
9	3.30	15.0	33.33 ± 6.73	30.59
10	3.30	15.0	34.45 ± 1.94	30.59
11	3.30	15.0	30.01 ± 4.81	30.59
12	3.30	15.0	23.33 ± 4.89	30.59
13	3.30	15.0	28.97 ± 4.12	30.59
DP	3.05	25.0	29.40 ± 3.58	28.98

**Note:** Standard deviation obtained from quadratic model for predicted value was 3.57. DP = de-esterified pectin.

**Table 4:** Analysis of variance (ANOVA) for the quadratic model.

Source	Sum of Squares	Mean Square	*p*-Value
Model	3887.42	777.48	<0.0001
A-Temperature	1328.37	1328.37	<0.0001
B-Vol of NaOH	1622.89	1622.89	<0.0001
AB	48.51	48.51	0.0923
A^2^	316.84	316.84	0.0016
B^2^	676.48	676.48	0.0002
Residual	89.37	12.77	
Lack of Fit	44.64	14.88	0.3819
Pure error	44.73	11.88	

**Table 5:** The mechanical properties of the cLMP and DP films with and without clindamycin HCl.

Films	Puncture Strength(N/mm^2^)	Elongation (%)	Young’s Modulus (N/mm^2^)	Thickness by Thickness Gauge (mm)	Thickness by SEM (µm)	Clindamycin HCl Content (%)
cLMP	13.72 ± 3.19 ^a^	2.75 ± 0.68 ^a^	67.69 ± 12.26 ^a^	0.048 ± 0.011 ^a^	~45	-
DP	11.13 ± 1.92 ^a^	2.52 ± 0.85 ^a^	61.79 ± 8.32 ^a^	0.049 ± 0.010 ^a^	~45	-
cLMPC	6.78 ± 1.85 ^b^	2.46 ± 0.29 ^a^	47.32 ± 3.35 ^b^	0.051 ± 0.007 ^a^	~43	93.47 ± 4.71 ^a^
DPC	4.66 ± 0.64 ^b^	2.77 ± 0.44 ^a^	44.77 ± 4.45 ^b^	0.048 ± 0.006 ^a^	~43	98.79 ± 2.69 ^a^

The experiment was performed in triplicate. Mean ± S.D. Values in the same column with different letters (a–b) indicate a significant difference (*p* < 0.05). Values in the same column superscripted with the same letters (a–b) indicate a non-statistically significant difference (*p* > 0.05).

**Table 6:** The antibacterial activity of pectin films containing clindamycin HCl against *Staphylococcus aureus* and *Cutibacterium acnes.*

Film Formulation	Zone of Inhibition (Diameter in mm)	
	*S. aureus*	*C. acnes*
Clindamycin solution (containing clindamycin HCl equivalent to clindamycin base 1%)	42.02 ± 0.52 ^a^	77.18 ± 1.50 ^a^
Blank film	7.18 ± 0.00 ^b^	7.63 ± 0.00 ^b^
cLMPC (cLMP film containing clindamycin HCl equivalent to clindamycin base 1%)	41.11 ± 0.33 ^a^	76.30 ± 0.98 ^a^
DPC (DP film containing clindamycin HCl equivalent to clindamycin base 1%)	40.78 ± 0.37 ^a^	74.04 ± 1.12 ^a^

The experiment was performed in triplicate. Mean ± S.D. Values in same column with different letters (a–b) indicate a significant difference (*p* < 0.05). Values in same column superscripted with same letters (a–b) indicate a non-statistically significantly difference (*p* > 0.05).
